# IgG4‐related lung disease progressing to respiratory failure

**DOI:** 10.1002/rcr2.641

**Published:** 2020-08-11

**Authors:** Yusuke Kunimatsu, Nozomi Tani, Izumi Sato, Yuri Ogura, Kazuki Hirose, Takayuki Takeda

**Affiliations:** ^1^ Department of Respiratory Medicine Japanese Red Cross Kyoto Daini Hospital Kyoto Japan

**Keywords:** Aortitis, corticosteroid, IgG4‐related lung disease, respiratory failure, reversibility

## Abstract

Immunoglobulin G4 (IgG4)‐related disease (IgG4‐RD) is a systemic immune‐mediated, fibroinflammatory disorder categorized as proliferative or fibrotic depending on the responsiveness of corticosteroid treatment. IgG4‐related lung disease (IgG4‐RLD) accounts for 13–14% of IgG4‐RD cases, but respiratory failure is quite rare. A 71‐year‐old man diagnosed with interstitial lung disease was referred to our department after a 10‐month observational period. He presented with respiratory failure at the first visit, with significant elevations in serum IgG4 levels and histopathological findings meeting the criteria of IgG4‐positive plasma cells and IgG4/IgG‐positive plasma cell ratio in transbronchial lung biopsy and inguinal lymph node biopsy, resulting in a diagnosis of IgG4‐RD. Systemic corticosteroid treatment promptly ameliorated the respiratory failure. ^18^F‐fluorodeoxyglucose (FDG) positron emission tomography/computed tomography showed significant FDG accumulation in the lung fields, indicating the proliferative and reversible status of IgG4‐RLD, which responded well to corticosteroid treatment. The patient recovered from respiratory failure even after a 10‐month observational period.

## Graphical abstract

Chest computed tomography (CT) showed non‐segmental reticular shadow and ground‐glass opacity predominantly spreading over the lymphatic tract, including the centrilobular and perilymphatic areas. ^18^F‐fluorodeoxyglucose (FDG) positron emission tomography/CT showed significant FDG accumulation in the lung fields, indicating the proliferative and reversible status of immunoglobulin G4‐related lung disease (IgG4‐RLD).

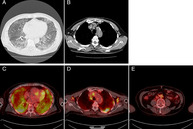

## Introduction

Immunoglobulin G4 (IgG4)‐related disease (IgG4‐RD) is a systemic immune‐mediated, fibroinflammatory, and multi‐organ disorder characterized by similar histopathological features to a dense infiltration of IgG4‐positive plasma cells in the affected organs [1]. An elevated blood serum IgG4 level is also a typical feature of IgG4‐RD [1]. IgG4‐RD potentially affects every organ [1, 2] and can be differentiated into proliferative and fibrotic subtypes depending on the responsiveness of corticosteroid treatment [1]. Corticosteroids are quite effective for the proliferative subtype and less effective for the fibrotic counterpart [3]. Lung involvement in IgG4‐RD, characterized as IgG4‐related lung disease (IgG4‐RLD), is observed in 13–14% of cases [4, 5, 6]. On the other hand, IgG4‐RLD resulting in respiratory failure is quite rare, which could be explained by its proliferative feature, although the timing of treatment as well as the observational period for IgG4‐RLD remain controversial. In this report, we describe a patient with IgG4‐RLD who developed respiratory failure 10 months after the first documented interstitial lung disease (ILD) and was successfully treated with systemic corticosteroids.

## Case Report

**Figure 1:**
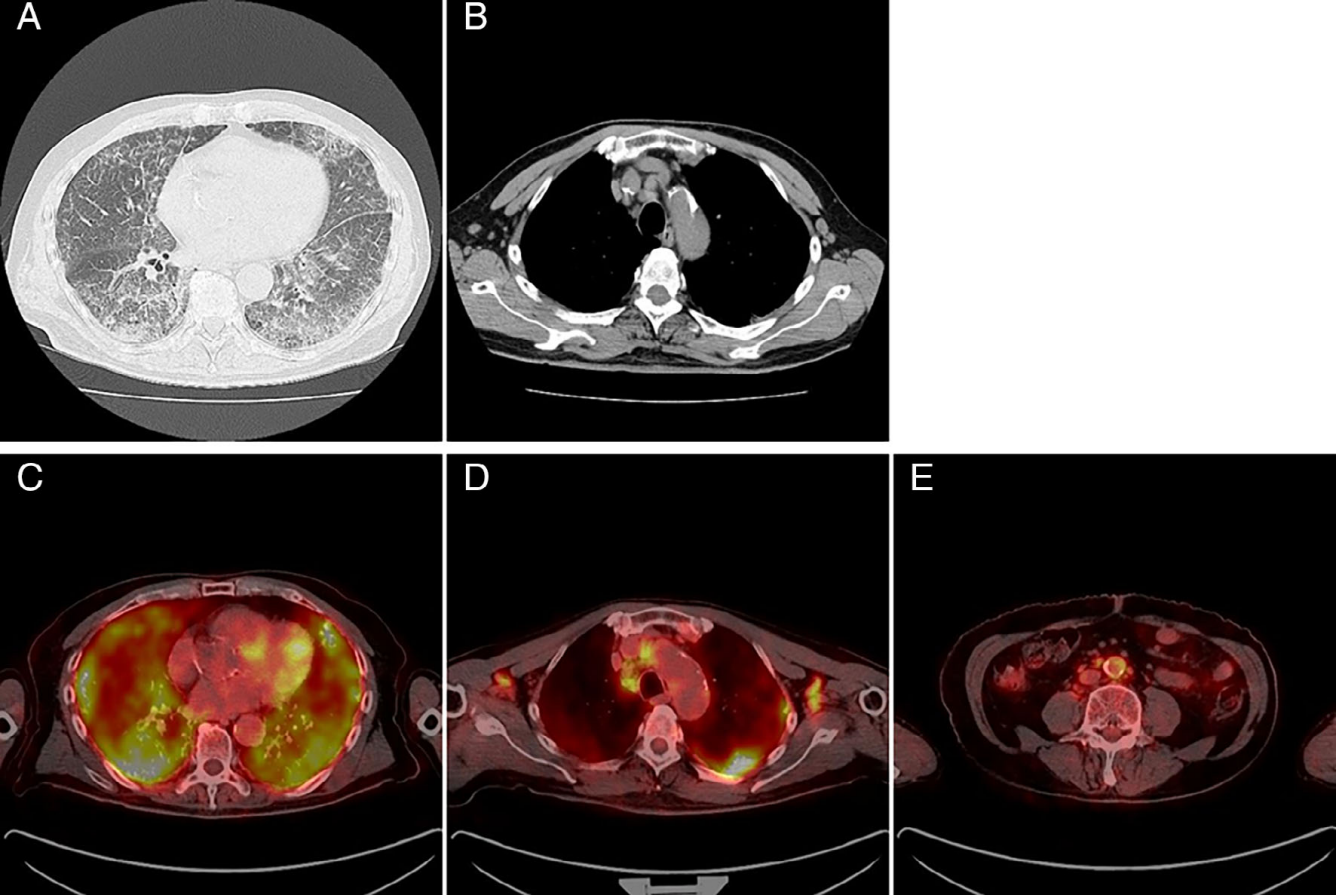
Chest computed tomography (CT) at the first consultation to our department showed non‐segmental reticular shadow and ground‐glass opacity predominantly spreading over the lymphatic tract, including the centrilobular and perilymphatic areas (A) accompanied by enlarged lymph nodes in the mediastinum and bilateral axillae (B). ^18^F‐fluorodeoxyglucose (FDG) positron emission tomography/CT showed significant FDG accumulation in the lung fields (C), the mediastinal, axillary (D), and para‐aortic (E) lymph nodes as well as abdominal aorta (E).

**Figure 2:**
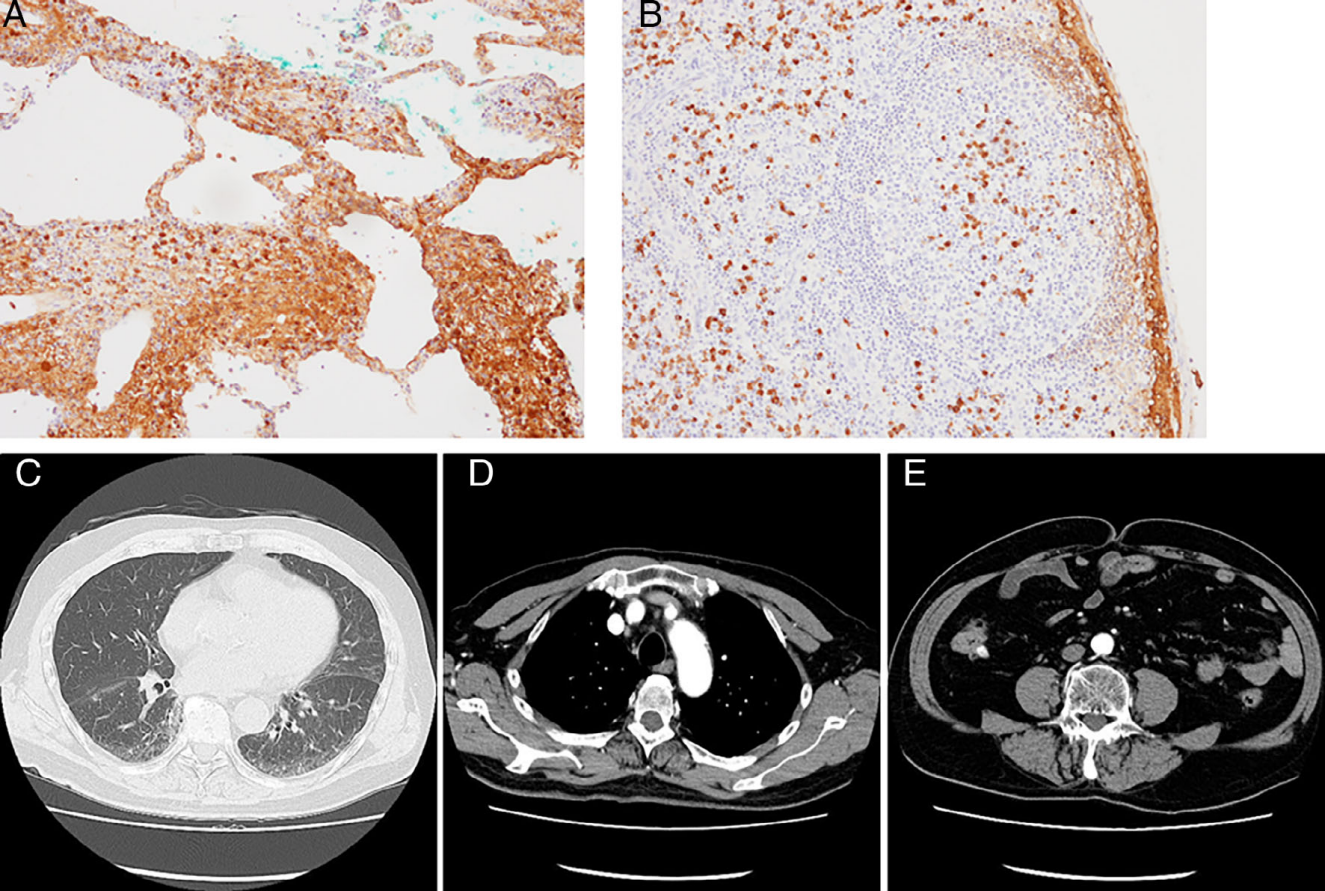
Specimen from transbronchial lung biopsy demonstrated lymphoplasmacytic infiltration with immunoglobulin G4 (IgG4)‐positive plasma cells >10/high‐power field (A). Inguinal lymph node biopsy elucidated IgG4‐positive plasma cell infiltration in lymphoid follicles with 10 cells/high‐power field and IgG4/IgG‐positive plasma cell ratio >40%, confirming the extrathoracic involvement (B). Magnification power was 200× each. Contrast‐enhanced computed tomography (CT) obtained four months after corticosteroid treatment showed substantial improvement in interstitial shadows (C) and shrinkage of the axillary, mediastinal (D), and para‐aortic (E) lymph nodes without wall thickening or luminal dilatation of abdominal aorta (E).

A 71‐year‐old male patient who had been diagnosed with ILD on chest computed tomography (CT) scans was referred to our department after a 10‐month observational period starting from the first consultation with the previous attending physician. Although the patient did not complain of dyspnoea, hypoxaemia was observed at the first consultation to our department, with peripheral capillary oxygen saturation (SpO_2_) and partial pressure of arterial oxygen (PaO_2_) at rest in room air scoring 89% and 59.5 mmHg, respectively. Chest CT showed progression of a non‐segmental reticular shadow and ground‐glass opacity (GGO) over 10 months, which predominantly spread over the lymphatic tract, including the centrilobular and perilymphatic areas (Fig. 1A), and enlarged lymph nodes were also observed in the mediastinum as well as bilateral axillae (Fig. 1B). CT findings were suggestive of lymphoproliferative disorder, carcinomatous lymphangitis, sarcoidosis, and IgG‐4RLD. Serum KL‐6 (Krebs von den Lungen‐6 antigen), SP‐D (surfactant protein D), and SP‐A (surfactant protein A) levels were elevated (778 U/mL, 214 ng/mL, and 28.2 mg/mL, respectively), with significant elevations in serum IgG and IgG4 levels (5002 and 2200 mg/dL, respectively) and no elevations in the levels of tumour markers or angiotensin‐converting enzyme. Lung function test revealed decreased diffusing capacity without a restrictive pattern; vital capacity (VC) 3.81 L (%VC: 94.3%), forced expiratory volume in 1 sec (FEV_1_) 3.13 L (FEV_1_%: 81.73%), diffusing capacity of the lung for carbon monoxide (DL_CO_) 7.65 mL/min/mmHg (%DL_CO_: 38.0%), and DL_CO_/alveolar volume (VA) 1.75 mL/min/mmHg/L (%DL_CO_/VA: 40.1%). ^18^F‐fluorodeoxyglucose (FDG) positron emission tomography (PET)/CT showed significant FDG accumulation in the lung fields (Fig. 1C), the systemic lymph nodes (Fig. 1D, E), including the cervical, hilar, mediastinal, axillary, para‐aortic, and inguinal regions, and the abdominal aorta (Fig. 1E). Bronchoalveolar lavage showed increased lymphocyte levels, and transbronchial lung biopsy demonstrated lymphoplasmacytic infiltration with IgG4‐positive plasma cells >10/high‐power field (Fig. 2A). Inguinal lymph node biopsy was performed to confirm extrathoracic involvement, which demonstrated IgG4‐positive plasma cell infiltration in lymphoid follicles with 10 cells/high‐power field and IgG4/IgG‐positive plasma cell ratio >40% (Fig. 2B). Systemic corticosteroid treatment with prednisolone at a dose of 40 mg/day (0.67 mg/kg) was quite effective, and the patient recovered from respiratory failure in 10 days with SpO_2_ scoring 96% in room air without desaturation on exertion. Then, prednisolone was tapered gradually. Contrast‐enhanced CT obtained four months after prednisolone treatment at a dose of 8 mg/day showed substantial improvement in interstitial shadows (Fig. 2C) and shrinkage of the axillary, mediastinal, and para‐aortic lymph nodes (Fig. 2D, E) without wall thickening or luminal dilatation of the abdominal aorta (Fig. 2E). The corticosteroid treatment is ongoing, and the patient has not shown recurrence.

## Discussion

IgG4‐RD was proposed as a novel clinical disease entity in 2011 [7], and comprehensive diagnostic criteria were established subsequently [8]. The initial criteria were as follows: (1) serum IgG4 elevation >135 mg/dL and (2) IgG4/IgG‐positive plasma cell ratio >40% as well as >10 cells/high‐power field of biopsy specimen [8], which was the basis of the recently updated three‐step classification process in 2019 with a specificity of 99.2% and a sensitivity of 85.5% [9].

IgG4‐RD has become widely recognized as a systemic inflammatory disease that affects multiple organs, including lacrimal glands, salivary glands, thyroid glands, pancreas, bile ducts, lungs, kidneys, aorta, and retroperitoneum [1, 8, 9]. While the treatment strategy for IgG4‐RD has not been fully established, systemic corticosteroid treatment has become standard and its response is typically good. However, in some cases resistant to corticosteroid, biologic agents, particularly a B cell‐depleting medication, or disease‐modifying anti‐rheumatic drugs are used in combination [1]. In the current case, the response to corticosteroid treatment was quite good and steroid‐sparing agents were not needed.

IgG4‐RLD affects the interstitium, mediastinum, airway, and pleura [10], with its clinical symptoms being related to the affected area, while 53% of patients are without pulmonary symptoms [11]. Although IgG4‐RLD affecting the interstitium is considered to result in respiratory failure when undiagnosed or untreated [1], the development of respiratory failure has never been reported. This could be explained by the proliferative feature of the lung involvement [1, 3]. However, the long‐term outcome of IgG4‐RLD has not been elucidated.

The long‐term outcomes of corticosteroid treatment in IgG4‐RD with the proliferative subtypes [1] such as salivary glands [12], pancreas [13, 14], and kidney [15, 16] were reported to be good. On the other hand, the efficacy of corticosteroids in the fibrotic subtype differs among patients according to the affected organ; no difference was observed in the response rate to corticosteroid between IgG4‐related and idiopathic retroperitoneal fibrosis [17], while corticosteroid treatment improved periaortic/periarterial lesions in most of the cases [18]. However, it is difficult to conjecture beforehand whether the disease is reversible in the affected organs in response to corticosteroid. Under these circumstances, early corticosteroid intervention is quite important in the management of IgG4‐RD [19].

The usefulness of ^18^F‐FDG PET/CT has been reported in the assessment of inflammatory disorder [20], including the activity of idiopathic interstitial pneumonia [21]. As ^18^F‐FDG PET/CT reflects inflammation, the accumulation of FDG could suggest the proliferative and reversible status of IgG4‐RLD. Therefore, the FDG accumulation could be a predictive biomarker of steroid responsiveness.

In the current case, FDG accumulation was observed in the lung fields, which could be the reason why these affected organs showed good response to systemic corticosteroid treatment even after a 10‐month observational period, showing reversibility in hypoxia. Although IgG4‐RLD progressing to respiratory failure is rare, accurate diagnosis of it as well as its detailed evaluation of reversibility and the responsiveness to corticosteroid are quite important. Appropriate early diagnoses and corticosteroid treatment are required in such case.

### Disclosure Statement

Appropriate written informed consent was obtained for publication of this case report and accompanying images.
